# Anxiety and Depression Traits Are Moderated by a Sex-Dependent Genetic Interaction Between 5-HTTLPR and BDNF

**DOI:** 10.3390/brainsci16080883

**Published:** 2026-08-19

**Authors:** G. Lorenzo Odierna, Christopher F. Sharpley, Vicki Bitsika

**Affiliations:** Brain-Behavior Research Group, University of New England, Armidale, NSW 2350, Australia; godierna@une.edu.au (G.L.O.); vicki.bitsika@une.edu.au (V.B.)

**Keywords:** 5-HTTLPR, BDNF, sex differences, anxiety, depression, anhedonia, psychological resilience

## Abstract

**Highlights:**

**What are the main findings?**

- The BDNF GA polymorphism has opposite effects depending on sex and 5-HTTLPR background.
- The interaction is selective for anxiety and anhedonic depression.

**What are the implications of the main findings?**

- Inverted vulnerability patterns between sexes underscore the potential importance of sex-specific approaches in psychiatric drug development and clinical care.
- Research should target specific subtypes rather than broad diagnoses to uncover meaningful associations.

**Abstract:**

Background/Objectives: Depression and anxiety disorders exhibit significant clinical heterogeneity, which complicates diagnosis and treatment. This study investigated whether tripartite interactions between biological sex, the serotonin transporter gene promoter region (5-HTTLPR), and brain-derived neurotrophic factor (BDNF) G196A polymorphisms explain specific symptom-level variations. Methods: A community sample of 271 Australian adults was genotyped for common 5-HTTLPR (short/long) and BDNF (G/A) variants. Participants completed self-reported measures of anxiety (SAS), depression subtypes (SDS; clinical content scales), and psychological resilience (CDRISC). Morning salivary cortisol, BMI, and substance use were assessed as potential confounds. Statistical analyses utilised a three-way factorial ANCOVA to test for interactions on raw scores, controlling for age. Significant omnibus interactions were decomposed via planned stratified ANCOVAs within each 5-HTTLPR stratum. Results: The BDNF GA genotype exerted opposite effects on anxiety and anhedonia depending on sex and 5-HTTLPR background. In females, GA was associated with increased symptoms on an ss background, whereas in males, GA was associated with increased symptoms on an ll background. These effects were highly specific to anxiety and anhedonic depression, while depressed mood, cognitive, and somatic subtypes remained unaffected. Findings were independent of cortisol, BMI, smoking, alcohol use, and psychological resilience. Conclusions: The findings reveal a complex, sex-dependent genetic interaction that selectively influences anhedonia and anxiety. They should be interpreted in the context of modest subgroup sizes following genotype stratification and require replication in larger independent cohorts. Nonetheless, this study highlights the value of considering sex-stratified genetic backgrounds and symptom specificity to advance personalised precision medicine in psychiatry.

## 1. Introduction

Depression and anxiety disorders represent major public health challenges, with substantial heterogeneity in their clinical presentation, underlying neurobiological pathways, and treatment models [1,2,3,4,5]. Understanding the cellular and molecular underpinnings of this heterogeneity is critical for developing more targeted therapeutic approaches to these conditions, often referred to as ‘precision medicine’ [6]. One potential avenue to identifying the patient-specific parameters of the heterogeneity is to leverage the growing body of knowledge describing the genetic architecture of depression and anxiety [7,8], with an appreciation for the highly multifactorial nature of genetic interactions.

Among the most extensively studied genetic variants in psychiatric research are polymorphisms in the serotonin transporter (5-HTT) gene promoter region (5-HTTLPR; reviewed in [9]), particularly the 43-base pair indel. The short (s) allele of 5-HTTLPR is associated with reduced transcriptional efficiency compared to the long (l) allele, resulting in decreased serotonin transporter expression [10,11] and, assumedly, bioavailable serotonin [12]. While early studies suggested a straightforward relationship between the s allele and increased risk for depression and anxiety [13,14,15], subsequent research has revealed a more nuanced picture. Some meta-analyses have revealed considerable heterogeneity in the association between 5-HTTLPR genotype and psychiatric outcomes, claiming that no meaningful relationship is detectable [16,17,18]. Others suggest 5-HTTLPR genotype acts as a conditional modulator of vulnerability that interacts with other genetic and environmental factors, such as life stress, in a temporally specific manner [19,20,21].

Another widely studied variant is in the brain-derived neurotrophic factor (BDNF) gene, notably the G196A polymorphism (rs6265; often referred to as Val66Met; reviewed in [22]). BDNF plays a crucial role in neuronal survival, differentiation, and synaptic plasticity [23]. The A allele (and consequent Val66Met amino acid substitution) results in impaired activity-dependent secretion of BDNF [24], potentially compromising the neuroplastic responses necessary for emotional regulation and stress adaptation. Carriers of the A allele are often reported to have an increased risk of a number of neuropsychiatric conditions, including depression [25], anxiety [26], bipolar disorder [27], and anorexia nervosa (among individuals with borderline personality disorder [28]). As with 5-HTTLPR polymorphisms, however, the influence of BDNF relies on multifactorial interactions between genes and the environment. For example, the influence of the G196A BDNF polymorphism on depression is dependent on stress associated with traumatic life events and childhood adversity [22]. 5-HTTLPR and BDNF polymorphisms can also genetically interact to moderate (1) depression in individuals who experienced childhood sexual abuse [29]; (2) teenage delinquency in individuals with monoamine oxidase polymorphisms [30]; and (3) postpartum depression in women who delivered children in autumn and winter [31]. As such, it is becoming increasingly clear that 5-HTTLPR and BDNF polymorphisms must be studied alongside other factors to accurately identify their influence on neuropsychiatry. One of those possible factors is the sex of individuals, with an established greater risk for developing anxiety [32] and depression [33] exhibited by women.

There are also ‘personality’ factors that influence the prevalence and effects of anxiety and depression, principally via the individual’s ability to cope with stress. One of these is psychological resilience [34], which is characterised as a personal trait or attribute that promotes rebounding from disappointments [35], being able to positively adjust to adverse circumstances [36], and adapting to challenging stressors [37]. Psychological resilience has been shown to act as a buffer between the experience of a traumatic event and the development of depression or anxiety [38] and to help individuals overcome the experience of trauma during early childhood and to progress to normal and satisfying lives [39,40]. As well as being conceptualised as a psychological variable, psychological resilience also has a biological character, based upon plasticity of the reward and fear circuits in the brain [41,42]. Additionally, psychological resilience has been described as “largely genetically determined” [43] based upon data from 2638 adolescent twins. Psychological resilience has been demonstrated to interact with 5-HTTLPR as a buffer between stress and depression [44].

Candidate gene studies of psychiatric traits have faced criticism regarding statistical power, replicability, and the potential for measurement artefacts to produce spurious interactions [17,45,46]. Addressing these concerns requires studies that investigate the interactions between multiple factors applying rigorous analytical approaches, including omnibus interaction models, sensitivity analysis and transparent reporting of effect size uncertainty. The present study aimed to examine the tripartite interaction between sex, 5-HTTLPR genotype, and BDNF genotype on measures of anxiety and depression subtypes in a community sample. We hypothesised that the effects of BDNF genotype on psychiatric outcomes would differ between males and females in a manner dependent on 5-HTTLPR background.

## 2. Materials and Methods

### 2.1. Participants

In this cross-sectional study, a community sample consisting of both depressed and non-depressed participants was recruited. The sample consisted of volunteers from one of the Australian Electoral Office electorates covering a large rural land area in northern New South Wales, who were invited by letter to participate in a study “about your mental health”. The letter emphasised that individuals who had not experienced mental health problems were welcome as much as those who had such experiences so that comparative data could be collected. Inclusion criteria were that participants were to be at least 18 years of age. Exclusion criteria were the presence of an acute medical illness. No other criteria were applied in order to reduce this study’s bias. A total of 271 individuals were recruited for this study, all of whom were genotyped. Participant numbers per genotype and their associated psychometric assessment data can be found in Table A1.

This project was approved by the University of New England Human Research Ethics Committee, Australia (Approval No. HE14–051). All participants gave written consent to this study. This study was conducted in accordance with the Declaration of Helsinki.

### 2.2. Genotyping

#### 2.2.1. DNA Extraction

Genomic DNA was extracted from mouthwash samples using a Gentra Puregene Buccal cell kit (Qiagen, Germantown, Maryland, USA). Mouthwash samples were centrifuged for 5 min at 2000× *g* to pellet cells, and DNA was extracted following the manufacturer’s protocol. The presence of intact, high molecular weight DNA was confirmed via gel electrophoresis and quantified using a NanoDrop 8000 spectrophotometer (NanoDrop, Wilmington, DE, USA).

#### 2.2.2. 5-HTTLPR Genotyping

5-HTTLPR genotyping for short and long alleles was carried out via polymerase chain reaction, as previously described [47]. The 5-HTTLPR alleles were amplified using the following primers: forward 5′ TCCTCCGCTTTGGCGCCTCTTCG 3′; reverse 5′ TGGGGGTTGCAGGGGAGATCCTG 3′. These primers yielded a 469 bp product for the short allele and a 512 bp product for the long allele. Each PCR reaction consisted of 1 × MyTaq Mix (Bioline, London, UK), 200 nM each primer, and 50 ng genomic DNA to obtain a final volume of 25 μL. The reaction was performed under the following conditions: 15 min denaturation at 95 °C, followed by 35 cycles of 95 °C (30 s), 65.5 °C (90 s) and 72 °C (60 s), followed by a final elongation step at 72 °C for 10 min. A total of 10 μL of each PCR product was loaded on a 3% agarose gel containing GelRed (Biotium, Fremont, CA, USA), run for 1 h at 100 V in TBE, and visualised under UV light. All genotyping reactions were completed in duplicate, with negative controls included in each assay.

#### 2.2.3. BDNF Genotyping

Genotyping for the BDNF G196A polymorphism was carried out by restriction fragment length polymorphism (RFLP) analysis, as previously described [48]. The BDNF polymorphic region was amplified using the following primers: forward 5′ ACTCTGGAGAGCGTGAAT 3′, reverse 5′ ATACTGTCACACACGCTC 3′. Each PCR reaction consisted of 1× MyTaq Mix (Bioline, UK), 250 nM each primer, and 50 ng genomic DNA to obtain a final volume of 25 μL. The reaction was performed under the following conditions: 5 min denaturation at 95 °C, followed by 35 cycles of 95 °C (30 s), 57 °C (45 s), and 72 °C (45 s), followed by a final elongation step at 72 °C for 10 min. A total of 10 µL of the resulting 308 bp PCR product was digested with FastDigest Hin1II, an isoschizomer of NlaIII (Thermo Scientific, Waltham, MA, USA), at 37 °C for 10 min. A total of 10 µL of each digest was loaded on a 3% agarose gel containing GelRed (Biotium, USA), run for 1 h at 100 V in TBE, and visualised under UV light. Digestion results in the following fragment sizes: AA: 168 + 75 bp; GA: 243 + 168 + 75 bp; and GG: 243 bp. All genotyping reactions were completed in duplicate, with negative controls included in each assay.

### 2.3. Questionnaires

#### 2.3.1. Background Questionnaire

Variables measured in this section included participants’ age and sex, ethnicity, weight, height, smoking habits, drinking habits, and medication use (anti-anxiolytics and antidepressants).

#### 2.3.2. Depression Questionnaire

The Self-rating Depression Scale (SDS) [49] is a 20-item self-report scale based on the Diagnostic Criteria and Associated Features of Major Depressive Disorder [50]. Participants are invited to indicate the level of severity of their experience of these 20 items during the last two weeks, and these responses provide a raw score ranging from 20 to 80. Zung [51] defined raw scores of 40 or more as indicative of “clinically significant depression”. The SDS has demonstrated split-half reliability of between 0.79 [52] and 0.94 [53] and internal consistency (alpha) of 0.88 for depressed and 0.93 for non-depressed individuals [54].

#### 2.3.3. Anxiety Questionnaire

The Self-rating Anxiety Scale (SAS) [55] consists of 20 items based on definitions of anxiety in the DSM criteria and drawn from DSM-5-TR Diagnostic Criteria for Generalised Anxiety Disorder [50]. Total raw scores range from 20 to 80, with low scores indicative of low anxiety. The SAS correlates at 0.75 with the Hamilton Anxiety Scale and has been shown to significantly discriminate between normal adult samples and patients with anxiety disorders [55,56]. The internal consistency (alpha) of the SAS is 0.82 [57]. Zung set a cutoff point raw score of 36, above which he described participants as having anxiety that was clinically significant [55].

#### 2.3.4. Psychological Resilience Questionnaire

Psychological resilience was assessed by the Connor–Davidson Resilience Scale (CDRISC) [58]. The CDRISC includes 25 items such as “I like a challenge”, “When things look hopeless, I don’t give up”, “I bounce back after illness or hardship”, and “I am able to adapt to change”. Total scores on the CDRISC range from 0 to 100 and are significantly correlated (0.83) with total scores on the Kobasa Hardiness Measure and negatively correlated with total scores on the Perceived Stress Scale (−0.76), indicating high concurrent validity. The CDRISC has acceptable reliability, ranging from 0.89 (Cronbach’s alpha) to 0.87 (test-retest reliability), and has been reported as having a 5-factor structure [58].

### 2.4. Cortisol Collection and Measurement

Participants provided a sample of their saliva using a Salivette^TM^ (SARSTEDT, Nümbrecht, Germany). Written and diagrammatic instructions were given to participants for this process, to be completed 45 min after waking in the morning and with no consumption of food or drink or smoking during that period.

Cortisol in saliva was measured using a specific salivary cortisol ELISA kit from Abnova Corporation (KA1885, Taipei, Taiwan). This is a solid-phase ELISA using a polyclonal rabbit antibody directed against cortisol. The assay is based on the principle of competitive binding, and endogenous cortisol in the sample competes with a cortisol–horseradish peroxidase conjugate for binding to the antibody. This ELISA has an intra-assay variability of 8.27% and inter-assay variability of 8.33%, with a spiking recovery of 100% and calibration range of 0.1–30 ng/mL. Salivary cortisol was assayed according to the manufacturer’s instructions. Briefly, 50 µL of neat saliva from participants or cortisol standards (0.1–30 ng/mL) were transferred to the appropriate wells of the 96-well microtiter plate. A total of 50 µL of enzyme conjugate was dispensed into each well with thorough mixing. The plate was incubated at room temperature with gentle rocking for 60 min. The contents of each well were aspirated, followed by 4 rinses with 300 µL wash solution. Substrate (200 µL) was added to each well, and the plate was incubated at room temperature for 30 min. The reaction was stopped with the addition of 50 µL stop solution, and absorbance was read at 450 nm immediately. All standards, controls and samples were assayed in duplicate, and results were calculated using a 4-parameter logistics curve fit.

A total of 234 participants volunteered to provide salivary cortisol samples (provision of saliva for cortisol analysis was not mandatory). Variation in the number of valid cortisol measurements across subgroups accounts for differences in degrees of freedom between cortisol and psychometric ANCOVAs reported in the results.

### 2.5. Data Handling and Statistical Analyses

Hardy–Weinberg equilibrium (HWE) was assessed for both polymorphisms using chi-squared goodness-of-fit tests. For the BDNF polymorphism, HWE was tested on the full recruited sample (N = 271, including the AA homozygotes) prior to any exclusions.

A total of 11 individuals homozygous for the BDNF AA genotype were excluded from the primary analysis due to insufficient sample size for meaningful statistical comparison across sex and 5-HTTLPR strata. The exclusion effectively models the A allele as having a dominant effect (GG vs. GA), which was necessitated by sample size constraints rather than a priori biological assumptions. Descriptive statistics for the excluded AA individuals are reported in Table A1 to allow qualitative assessment of consistency with the dominant model.

The primary statistical analysis employed a three-way factorial ANCOVA with sex (male, female), BDNF genotype (GG, GA), and 5-HTTLPR genotype (ss, sl, ll) as fixed factors and age as a covariate. This model was applied to each outcome measure: anxiety (SAS total score), four clinical content depression subtypes (depressed mood, anhedonia, somatic depression, and cognitive depression), psychological resilience (CDRISC total), morning salivary cortisol (log-transformed due to the well-established right-skewed distribution of salivary cortisol) and body mass index (BMI). Sample sizes for individual analyses vary due to missing data on specific measures and are reflected in the degrees of freedom reported for each ANCOVA. The number of participants with valid data (after removing AA individuals) were as follows: SAS n = 239, SDS subscales = 243, CDRISC n = 244, cortisol n = 234, and BMI n = 210. Full results from this analysis can be found in Table A2.

For outcomes showing a significant omnibus three-way interaction, planned follow-up analyses were conducted by testing the interaction between sex and BDNF genotype within each 5-HTTLPR stratum (ss, sl, ll) separately using ANCOVA with age as a covariate. These stratified analyses decompose the significant omnibus interaction into its constituent patterns and are treated as planned contrasts rather than independent exploratory tests. To address concerns regarding multiple comparisons [45], Benjamini–Hochberg false discovery rate (FDR)-adjusted *p*-values were calculated across all tests. The Benjamini–Hochberg FDR correction was applied at two levels: (1) across the eight omnibus three-way interaction tests (one per outcome) to control for multiple outcome testing, and (2) across the 24 stratified follow-up ANCOVAs to control for multiple comparisons within the decomposition of significant interactions. Given that the stratified analyses are planned decompositions of significant omnibus interactions, the nominal *p*-values are reported in the main text with FDR-adjusted values provided in Table A3 for transparency.

Several sensitivity analyses were conducted to assess the robustness of significant findings: (1) leave-one-out analysis, in which each participant was sequentially removed and the ANCOVA re-run to assess whether findings were driven by individual observations in small subgroups; (2) rank-transformed analysis, in which outcome scores were replaced by their ranks prior to ANCOVA, to assess whether findings were robust to violations of distributional assumptions and to address concerns about scale-property artefacts [46]; (3) exclusion of participants reporting current use of anxiolytic or antidepressant medication, to assess potential confounding by psychotropic medication; and (4) bootstrapped 95% confidence intervals (5000 iterations) for partial eta-squared effect sizes (η^2^p), to honestly communicate effect size uncertainty given the modest subgroup sizes [17]. The η^2^p was calculated for all ANCOVA effects. Associations between smoking/alcohol consumption and genotype were examined using chi-squared tests of independence for binary variables and Kruskal–Wallis tests for ordinal alcohol consumption levels. All statistical tests were two-tailed with α set to 0.05.

Data handling, data analysis and graph generation were conducted in Python using pandas v2.2.2 [59], Matplotlib v3.9.2 [60], NumPy v2.3.4 [61], SciPy v1.13.1 [62], statsmodels v0.14.4 [63] and seaborn v0.13.2 [64] via Jupyter Notebook v7.4.5 [65]. The code used to analyse this data is available online at https://github.com/LorenzoOdierna/AnxDepStudy5-HTTLPRBDNFSex.

## 3. Results

### 3.1. Demographic Information

A total of 271 individuals were recruited for this study, all of which were genotyped for BDNF and 5-HTTLPR polymorphisms. With respect to the genotype distribution of the 5-HTTLPR 43 base pair deletion polymorphism within the full sample of 271 individuals, 52 (19.2%) were ss homozygous, 148 (54.6%) were sl heterozygous, and 71 (26.2%) were ll homozygous. With respect to the genotype distribution of the BDNF rs6265 G196A polymorphism within the full sample of 271 individuals, 177 (65.3%) were GG homozygous, 83 (30.6%) were GA heterozygous, and 11 (4.1%) were AA homozygous. Both the 5-HTTLPR and BDNF genotype distributions were in Hardy–Weinberg equilibrium (5-HTTLPR: χ^2^(1) = 2.58, *p* = 0.11, allele frequencies of s = 0.47, l = 0.53; BDNF: χ^2^(1) = 0.1, *p* = 0.75, allele frequencies of G = 0.81, A = 0.19), supporting the reliability of the genotyping procedures. These genotype distributions are largely consistent with previous reports for populations with considerable European descent [66,67,68,69]. After exclusion of AA individuals or those who did not provide valid age data, a sample of 254 remained. Within this sample, there was no significant difference in mean age of the individual genotype groupings (*F*(5, 248) = 1.47, *p* = 0.2), including GG; ss (55.3 ± 21.1), GA; ss (53.8 ± 16.6), GG; sl (53.9 ± 17.6), GA; sl (58.0 ± 15.3), GG; ll (51.0 ± 17.4), and GA; ll (46.6 ± 11.6). Additional demographic information regarding participants used in this study can be found in Table 1.

### 3.2. Sex-Dependent Effects of Genotype on Self-Rated Anxiety

A three-way factorial ANCOVA (Sex × BDNF × 5-HTTLPR genotype + Age) revealed a significant three-way interaction for SAS total score (F(2,222) = 4.5, *p* = 0.012, η^2^p = 0.039; FDR-corrected *p* = 0.048). No main effects of BDNF genotype or 5-HTTLPR genotype were significant (*p* = 0.37 and *p* = 0.78, respectively) and the Sex × BDNF and Sex × 5-HTTLPR two-way interactions were non-significant (*p* = 0.7 and *p* = 0.98, respectively). Age was a significant covariate (F(1,222) = 13.05, *p* < 0.001, η^2^p = 0.056).

**Figure 1:**
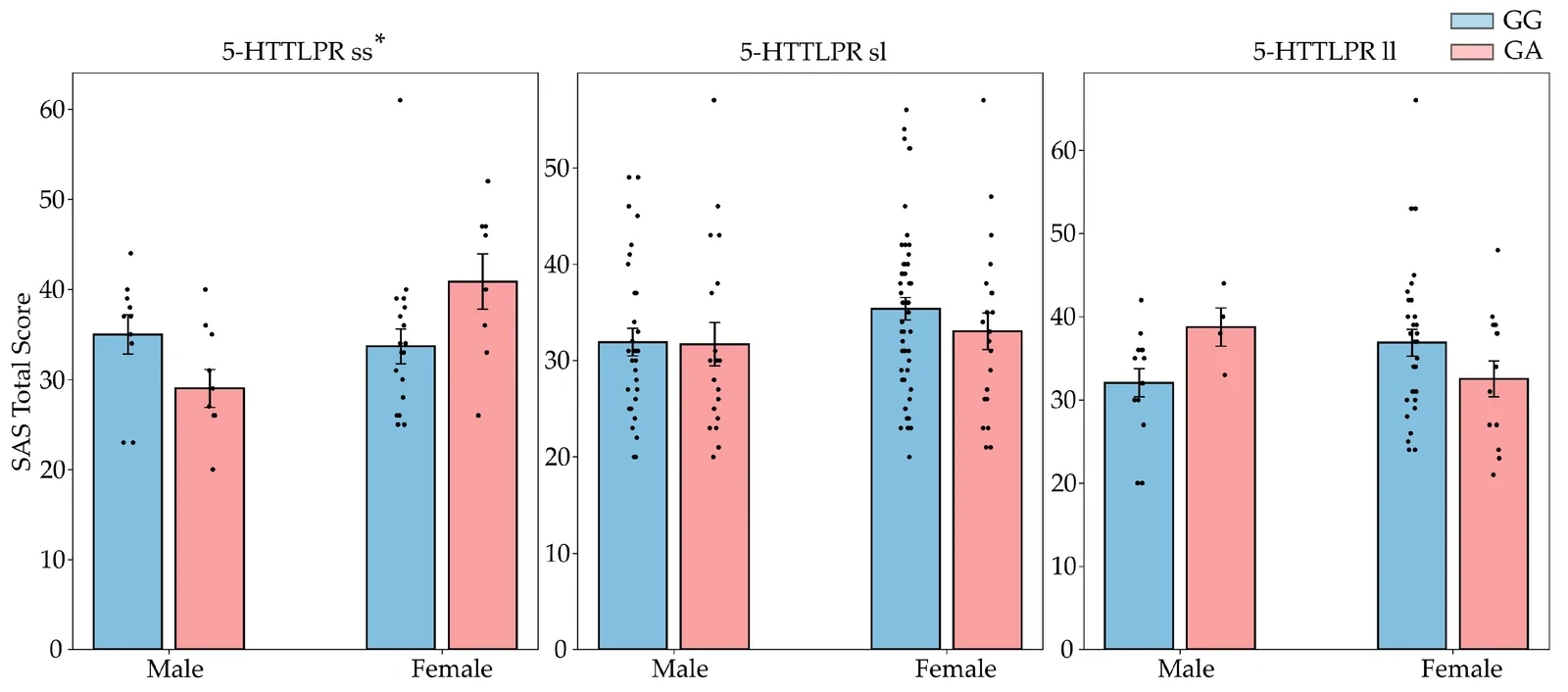
Sex-dependent interaction between 5-HTTLPR and BDNF polymorphisms on anxiety. Bar plots showing total Zung Self-rated Anxiety Score (SAS) across different genotypes of 5-HTTLPR and BDNF, grouped by sex. 5-HTTLPR polymorphisms include the ‘short’ (s) and ‘long’ (l) alleles. BDNF polymorphisms include the ‘G 196’ and ‘A 196’ alleles. ANCOVA was applied to detect statistically significant interactions between sex and BDNF genotype within each 5-HTTLPR genotype stratum. Black circles represent data from individual participants. *, *p* < 0.05.

To decompose the significant three-way interaction, planned follow-up ANCOVAs tested the Sex × BDNF genotype interaction within each 5-HTTLPR stratum. A significant Sex × BDNF interaction was observed in ss carriers (F(1,42) = 5.12, *p* = 0.029, η^2^p = 0.11) with a trend in ll carriers (F(1,57) = 3.65, *p* = 0.06, η^2^p = 0.06) and no interaction in sl carriers (F(1,121) = 0.6, *p* = 0.44, η^2^p = 0.005). Inspection of group means (Table A1; Figure 1) indicated that on an ss background, the GA genotype was associated with higher anxiety in females (GG µ = 33.7 ± 8.4; GA µ = 40.9 ± 8.7) and lower anxiety in males (GG µ = 35.0 ± 6.9; GA µ = 30.0 ± 8.3), whereas on an ll background, the GA genotype was associated with higher anxiety in males (GG µ = 32.1 ± 6.3; GA µ = 38.8 ± 4.6) and lower anxiety in females (GG µ = 36.9 ± 9.1; GA µ = 32.5 ± 8.2).

Leave-one-out sensitivity analysis for the ss stratum showed that 43 of 47 interactions (91.5%) remained significant at *p* < 0.05 (*p*-value range = 0.003–0.07; median *p* = 0.032), indicating robust findings. Rank-transformed analysis confirmed the ss interaction (*p* = 0.019). The finding also remained significant after excluding participants on psychotropic medication (*p* = 0.026). Bootstrapped 95% confidence intervals for η^2^p in the ss stratum were [0.002, 0.397], reflecting substantial uncertainty in effect size magnitude given the modest subgroup sizes.

A major determinant of anxiety is the expression of glucocorticoids, which are directly linked with enhanced anxiety [70]. This is specifically true for cortisol levels at the time of awakening rather than at later times in the day [71]. To test whether the sex-dependent effect of BDNF and 5-HTTLPR genotype on anxiety was associated with elevated glucocorticoid expression, we measured morning salivary cortisol levels and tested whether this differed according to BDNF genotype, 5-HTTLPR and sex. Since average cortisol levels change considerably over the lifespan [72], an ANCOVA controlling for age was performed. The omnibus three-way ANCOVA revealed no significant three-way interaction for morning salivary cortisol (F(2,215) = 0.14, *p* = 0.87, η^2^p = 0.001). As such, sex-dependent differences in the genetic interaction between BDNF and 5-HTTLPR polymorphisms on anxiety cannot be confidently attributed to elevated awakening cortisol levels.

### 3.3. Sex-Dependent Effects of Genotype on Clinical Content Subtypes of Depression

5-HTTLPR and BDNF polymorphisms have previously been investigated in the context of depression, where both have been found to be associated with depression risk [19,22,73]. This interaction is sex-dependent such that females are more vulnerable to the effects of the BDNF GA allele, moderated by the 5-HTTLPR background [74]. To explore how the tripartite interaction between sex, BDNF genotype and 5-HTTLPR genotype influences depression beyond what has been previously reported at a broad and agranular level, we aimed to investigate subtypes of depression. To achieve this, we measured subscales of the SDS that report on four previously validated ‘clinical content’ subtypes of depression, defined as ‘depressed mood’, ‘anhedonic depression’, ‘cognitive depression’ and ‘somatic depression’ [75]. These four subtypes are differentially associated with measures of work-related stress [76] and display distinct electroencephalographic signatures [77]. An omnibus three-way ANCOVA revealed a significant three-way interaction for anhedonic depression (F(2,226) = 5.76, *p* = 0.004, η^2^p = 0.049; FDR-corrected *p* = 0.029), but not for depressed mood (F(2,227) = 1.05, *p* = 0.35, η^2^p = 0.009), somatic depression (F(2,227) = 0.16, *p* = 0.85, η^2^p = 0.001), or cognitive depression (F(2,227) = 1.19, *p* = 0.31, η^2^p = 0.01). Thus, the sex-dependent genetic interaction between BDNF and 5-HTTLPR polymorphisms that influences depression [74] does so specifically via traits relating to anhedonia.

**Figure 2:**
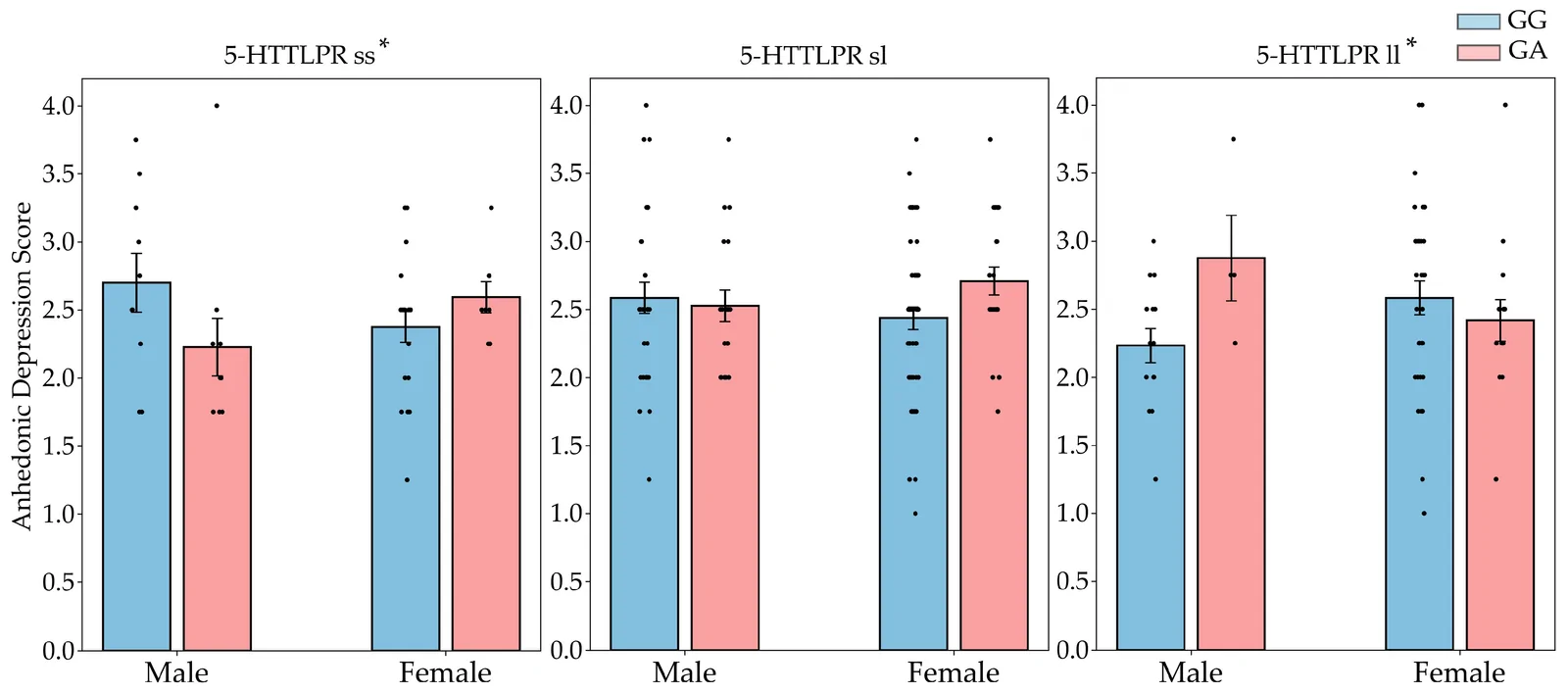
Sex-dependent interaction between 5-HTTLPR and BDNF polymorphisms on anhedonia. Bar plots showing subscale Zung Self-rated Depression Score for anhedonic depression across different genotypes of 5-HTTLPR and BDNF, grouped by sex. 5-HTTLPR polymorphisms include the ‘short’ (s) and ‘long’ (l) alleles. BDNF polymorphisms include the ‘G 196’ and ‘A 196’ alleles. ANCOVA was applied to detect statistically significant interactions between sex and BDNF genotype within each 5-HTTLPR genotype stratum. Black circles represent data from individual participants. *, *p* < 0.05.

Planned follow-up ANCOVAs for anhedonic depression revealed significant Sex × BDNF interactions in ss carriers (F(1,43) = 4.42, *p* = 0.042, η^2^p = 0.09) and ll carriers (F(1,58) = 4.06, *p* = 0.049, η^2^p = 0.065), but not in sl carriers (F(1,123) = 3.53, *p* = 0.063, η^2^p = 0.028). The pattern of the group means closely mirrored that observed for anxiety (Table A1; Figure 2): on an ss background, female GA carriers showed higher anhedonia scores than female GG carriers (GG µ = 2.4 ± 0.5; GA µ = 2.6 ± 0.3), while male GA carriers showed lower scores (GG µ = 2.7 ± 0.6; GA µ = 2.2 ± 0.7). On an ll background, the pattern reversed, with male GA carriers showing higher anhedonia (GG µ = 2.2 ± 0.5; GA µ = 2.9 ± 0.6) and female GA carriers showing lower scores (GG µ = 2.6 ± 0.7; GA µ = 2.4 ± 0.6).

Leave-one-out analysis for the ss stratum showed that 32 of 48 iterations (66.7%) remained significant at *p* < 0.05 (*p*-value range = 0.007–0.08; median *p* = 0.044), indicating robustness. For the ll stratum, 27 of 63 iterations (42.9%) remained significant (*p*-value range = 0.018–0.24; median *p* = 0.052), indicating sensitivity to individual observations, consistent with the low number of male GG; ll individuals (n = 4). Rank-transformed analyses confirmed both the ss and ll interactions (*p* = 0.02 and *p* = 0.043, respectively). After excluding medicated participants, both interactions were attenuated (*p* = 0.132 and *p* = 0.237); however, medication use did not differ across genotype groups (*p* = 0.707), suggesting that the attenuation reflects reduced statistical power from participant loss rather than a confounding effect. Bootstrapped 95% confidence intervals for η^2^p were [0.003,0.338] for ss and [0.001,0.232] for ll, reflecting substantial uncertainty in effect size magnitude.

### 3.4. Examination of Smoking and Drinking Across Genotype and Sex Subgroups

Depression and anxiety can be influenced by addiction and substance abuse, particularly in the context of alcohol and nicotine consumption [78,79]. As such, we considered the possibility that the sex-dependent effect of BDNF and 5-HTTLPR genotype on self-rated measures of anxiety and anhedonic depression might be mechanistically downstream of increased use of alcohol and nicotine. This was particularly important given known links between the serotonin signalling system and the types of impulsive behaviours that can promote addiction [80]. To explore such a connection, the rate of smoking and alcohol consumption in the community sample were examined. Application of a chi-squared test suggested that there was no meaningful difference in the proportion of smokers across all combinations of genotypes and sexes (χ^2^ = 16.88, *p* = 0.11; Table 2). The same was true for alcohol consumption, with there being no detectable difference in the proportion of individuals across all subgroups based on a range of categories representing increasing levels of alcohol consumption (χ^2^ = 10.81, *p* = 0.46; Table 2). As such, the observed sex-dependent differences in depression and anxiety measures due to BDNF and 5-HTTLPR genotype cannot be attributed to altered intake of commonly available substances of abuse or addiction.

### 3.5. Examination of Body Mass Index Across Genotype and Sex Subgroups

Incidence of depression is often associated with body mass [81]. Overweight and obese individuals report more regular or severe presentation of depression-associated symptoms [82]. The prevalence of anxiety disorders is also higher in obese individuals [83], with some data suggesting obesity is associated with an enhanced risk of clinically relevant anxiety [84]. One reasonable explanation for the sex-dependent effect of BDNF and 5-HTTLPR genotype on depression and anxiety might therefore be that they primarily influence body mass, which then results in enhanced expression of depression and anxiety symptoms. The role of muscle-secreted BDNF in modulating pancreatic insulin release could reasonably support such a mechanism [85]. To test this possibility, we investigated body mass index (BMI) in the community sample. A three-way ANCOVA with age as a covariate revealed that the three-way sex × BDNF × 5-HTTLPR interaction was not significant for BMI (F(2,193) = 0.477, *p* = 0.621, η^2^p = 0.005; Table 3), and no significant two-way interactions were observed between sex and BDNF (F(1,193) = 0.009, *p* = 0.926, η^2^*p* < 0.001), sex and 5-HTTLPR (F(2,193) = 1.794, *p* = 0.169, η^2^p = 0.018), or BDNF and 5-HTTLPR (F(2,193) = 0.142, *p* = 0.868, η^2^p = 0.002). This result indicates that the observed sex-dependent differences in measures of depression and anxiety due to BDNF and 5-HTTLPR genotype cannot be explained by potential upstream effects on body mass.

### 3.6. Sex-Dependent Effects of Genotype on Psychological Resilience

Vulnerability to anxiety and depression is strongly modulated by psychological resilience, which encompasses a range of qualities relating to an individual’s adaptability in response to stress [86,87]. There has been increasing recognition of the role played by polymorphisms in 5-HTTLPR and BDNF in the determination of psychological resilience [88,89], though the exact mechanism underlying these associations remains incompletely identified. To explore if sex, BDNF genotype and 5-HTTLPR genotype interact to impact depression and anxiety via modulation of psychological resilience, we measured Connor–Davidson Resilience Scale (CDRISC) scores within the community sample. The omnibus three-way ANCOVA for CDRISC total score did not reveal a significant three-way interaction (F(2,227) = 0.58, *p* = 0.562, η^2^p = 0.005). Exploratory stratified analysis showed no significant Sex × BDNF interaction in any 5-HTTLPR strata (ss: F(1,44) = 0.91, *p* = 0.346; sl: F(1,123) = 1.01, *p* = 0.317; ll: F(1,58) = 0.13, *p* = 0.725). As such, the tripartite interaction with respect to anxiety and anhedonia cannot be explained by differences in psychological resilience.

## 4. Discussion

The present study reveals the underlying complexity of the interaction between 5-HTTLPR and BDNF polymorphisms influencing anxiety and depression traits. Most notably, our findings suggest that this interaction is phenotype-specific and sex-dependent. We report that the BDNF GA genotype exerts opposite effects depending on both sex and 5-HTTLPR allelic background. In females, the GA genotype is associated with increased anxiety and anhedonic depression on an ss background, whereas in males, the GA genotype is associated with increased symptoms on an ll background. This pattern was not observed for other subtypes of depression, including depressed mood, cognitive depression, or somatic depression, indicating a high degree of specificity in the nature of the relationship between the genetic polymorphisms and psychiatric measures studied here.

### 4.1. Specificity of Genetic Effects to Anxiety and Anhedonic Depression

The selective impact of the interactions between BDNF and 5-HTTLPR genotype on anxiety and anhedonic depression, but not other depression subtypes, is particularly noteworthy. The presence of a three-way interaction specifically for anxiety and anhedonic depression but not for other SDS subscales and CDRISC-measured psychological resilience provides meaningful evidence against scale-property artefacts, which is an unignorable concern in psychopathology research that must be accounted for [46]. Such specificity is arguably in line with known underlying neurobiology, as anhedonia is a key symptomatic link between depression and anxiety [90]. This is particularly interesting when considering the neurobiological context, seeing as both anhedonia and anxiety involve disruptions to the ventral striatum [91,92], nucleus accumbens [93,94], and prefrontal cortex [95,96]. The serotonin system plays a critical role in reward prediction and processing [97], with expression of the serotonin transporter (5-HTT) directly influencing the availability of serotonin in synaptic spaces within reward-related circuits [12]. BDNF, in turn, is essential for the structural and functional plasticity of these circuits [23], particularly in response to stress and reward-related learning. The convergence of altered serotonergic tone (via 5-HTTLPR) and altered neuroplasticity (via BDNF Val66Met) may interact to specifically disrupt the neural mechanisms underlying reward sensitivity and threat appraisal, promoting anhedonia and anxiety specifically, rather than affecting other aspects of depressive symptomatology such as mood dysregulation, cognitive dysfunction or somatic issues.

The absence of genetic effects on depressed mood, cognitive depression, and somatic depression subtypes suggests these dimensions may be influenced by distinct genetic and neurobiological pathways. This finding aligns with emerging evidence that depression is not a unitary construct but rather comprises multiple symptom dimensions with partially independent aetiologies [75]. Our results support the utility of examining clinical content depression subtypes in genetic studies, as aggregating across all symptoms may obscure specific gene–symptom relationships.

### 4.2. Sex-Dependent and 5-HTTLPR-Dependent Directionality of BDNF Effects

Perhaps the most striking finding of this study is the opposite directionality of BDNF GA effects in males versus females depending on 5-HTTLPR genotype. On an ss background, females with the GA genotype showed elevated anxiety and anhedonic depression, whereas on an ll background, males with the GA genotype showed elevated symptoms. This pattern suggests that the functional consequences of the BDNF A allele are fundamentally modulated by both the serotonergic milieu (determined by 5-HTTLPR genotype) and sex-specific factors. We hypothesise that one potential explanation for this sex-dependent pattern involves oestrogen, which has been shown to upregulate the expression of both BDNF and 5-HTT [98,99]. In females, the constitutionally low 5-HTT expression caused by the ss genotype and the impairment in activity-dependent BDNF secretion caused by the BDNF GA genotype may create a “double hit” that could interact with fluctuating oestrogen levels over the menstrual cycle to specifically impair neural circuits underlying threat and reward assessment. In contrast, in males with the ll genotype, higher baseline 5-HTT expression may create a different serotonergic context in which the BDNF GA genotype exerts detrimental effects, though the exact nature of this context remains unclear. It must be noted that this interpretation is largely speculative in nature, as oestrogen levels, menstrual phase and menopausal status were not assessed in the present study and present important variables for future investigation. Most importantly, while the omnibus three-way interactions for anxiety and anhedonia were statistically significant and survived FDR correction, the individual stratified effects did not and thus should be interpreted as exploratory decompositions that characterise the pattern of the interaction rather than as independently confirmed findings.

### 4.3. Independence from Morning Cortisol Expression, Substance Use, Body Mass and Psychotropic Medication

Our finding that the sex-dependent genetic effects on anxiety and anhedonic depression are independent of morning cortisol levels is informative. Glucocorticoids are known to influence both anxiety symptoms and depressive behaviours [100,101]. The absence of genotype-related differences in cortisol suggests that the observed psychiatric effects are not mediated by gross alterations in awakening cortisol expression. However, this finding should be interpreted with caution, as cortisol expression varies substantially and we did not assess diurnal cortisol rhythm, stress-induced cortisol release, or glucocorticoid receptor sensitivity, any of which may reveal more subtle genotype-related differences.

Similarly, the absence of differences in smoking and alcohol consumption across genotype groups indicates that the observed psychiatric effects are not secondary to substance use patterns. This is an important control given the well-established bidirectional relationships between substance use and both depression and anxiety [78,79], as well as the known involvement of serotonergic signalling in impulsivity and addiction-related behaviours [80].

The lack of genotype-related differences in body mass index further strengthens the interpretation that the genetic effects on anxiety and anhedonic depression are direct rather than mediated through metabolic pathways. While BDNF does play a role in metabolic regulation, including modulation of pancreatic insulin secretion [85], our data suggest that the psychiatric effects of the sex-dependent interaction between BDNF and 5-HTTLPR operate through neural rather than metabolic mechanisms.

Lastly, psychotropic medication use did not differ significantly across genotype subgroups. This is an important consideration given that selective serotonin reuptake inhibitors directly modulate serotonin transporter function. Sensitivity analyses excluding all medicated participants showed that the anxiety findings remained significant while the anhedonia findings were attenuated below significance. Given the absence of any association between medication use and genotype, this attenuation likely reflects reduced statistical power from the smaller sample rather than confounding. Nevertheless, future studies should consider medication status as a stratification variable or exclusion criterion to more definitively disentangle genetic and pharmacological interactions.

### 4.4. Limitations of This Study

A primary limitation of this study is the modest sample size (N = 271). When stratified by sex, 5-HTTLPR genotype and BDNF genotype, several subgroups became quite small, which limits statistical power for detecting higher-order interactions and increases the risk of size effect inflation. The partial eta-squared values observed in this study are accompanied by wide bootstrapped 95% confidence intervals, indicating substantial uncertainty in effect size magnitude. Such large effect sizes in small samples are frequently inflated, a phenomenon known as the ‘winner’s curse’, and may not replicate at the same magnitude in larger cohorts [102]. The direction and specificity of the interaction pattern, rather than the precise effect size magnitudes, should be considered the primary finding of this study.

Due to insufficient sample size, the number of individuals homozygous for the BDNF AA rs6265 polymorphism was too low (and for some genotype groupings, completely absent). As such, the 11 AA individuals recruited for this study were excluded from the analysis presented here. The resultant selective comparison between GG and GA individuals effectively models the A allele as having a dominant effect. While this approach was necessitated by sample size constraints, it precludes testing for additive or recessive genetic models, which may more accurately reflect the true mode of inheritance. While our findings show statistically significant and biologically coherent patterns within the current dataset, they must be interpreted cautiously. This is especially relevant considering no nominal *p* values for within-strata interactions survived FDR correction (applied across 24 tests). The omnibus three-way interactions for anxiety and anhedonia both survived FDR correction, and the results were internally consistent and specific. This does provide some level of protection against false positives, alongside the convergence between the omnibus three-way interactions and the stratified follow-up analyses. To provide some context on the effects of our study’s sample size, a post hoc sensitivity power analysis was conducted using the actual unbalanced sample frequencies with the goal of determining the smallest effects detectable at 80% power (α = 0.05). For the three-way interaction, the minimum detectable η^2^p was 0.013, corresponding to a small-to-medium effect. The observed three-way interactions for anxiety (η^2^p = 0.039) and anhedonia (η^2^p = 0.049) both exceeded this threshold, indicating that this study was adequately powered to detect effects of the magnitude observed. For main effects and two-way interactions with one numerator degree of freedom, the minimum detectable effect was η^2^p = 0.017; for those with two numerator degrees of freedom, η^2^p = 0.013. It bears mentioning that though our study was well powered to detect small-to-medium effects in the omnibus model, the stratified follow-up analyses within individual 5-HTTLPR strata necessarily have reduced power due to smaller subgroup sizes. As such, replication in larger cohorts is necessary before highly confident conclusions can be drawn regarding these results.

Beyond the sample size, several additional limitations of the present study should be acknowledged. First, our sample consisted primarily of individuals of European descent, and the generalisability of our findings to other ethnic populations remains to be established. Given evidence that 5-HTTLPR and BDNF associations with psychiatric outcomes differ across ethnic groups [27,103], exploration of their effects in diverse populations is essential. Second, we examined only the G196A (Val66Met) polymorphism in BDNF, and the 5-HTTLPR insertion/deletion polymorphism was examined at a superficial level, not accounting for other identified sub-variants. Many other variants of 5-HTTLPR have been identified, such as the G and A alleles of the long insertion (lA and lG) or XL versions [9,104,105], many of which have been reported to influence psychiatric traits. Other functional variants in these genes, as well as interactions with additional genetic loci, may contribute to the observed phenotypes and thus should be considered in future work. Third, our assessment of cortisol was limited to morning sampling of saliva; more comprehensive assessment of cortisol, including diurnal rhythm and dexamethasone suppression testing, may reveal more subtle genotype-related differences. Fourth, our cross-sectional design precludes causal inference; longitudinal studies examining how these genetic interactions influence the development and course of anxiety and depression over time would be valuable. It is also worth reiterating that after excluding medicated individuals, the interactions for anhedonia were attenuated. Though we interpret this attenuation as occurring due to diminished statistical power from the reduction in sample size, it remains possible that medication status plays a significant role in the interaction for anhedonia. As such, replication in larger cohorts is required before confident conclusions can be drawn.

## 5. Conclusions

This study indicates a complex, sex-dependent interaction between 5-HTTLPR and BDNF polymorphisms that specifically influences anxiety and anhedonic depression. The BDNF GA genotype exerts opposite effects in males versus females depending on 5-HTTLPR allelic background, with females showing increased vulnerability on an ss background and males showing increased vulnerability on an ll background. These effects are independent of awakening cortisol levels, substance use, and body mass and show remarkable specificity for anxiety and anhedonic symptoms rather than affecting all dimensions of depression. While these findings are internally consistent and biologically coherent, the modest subgroup sizes investigated here necessitate cautious interpretation and replication in larger, pre-registered studies before clinical implications can be drawn. Nonetheless, this study underscores the importance of considering sex, genetic background, and symptom specificity in psychiatric genetics research.

## Figures and Tables

**Table 1:** Demographic data, including depression assessment metrics, by sex.

Characteristic	Total (N = 254)	Male (n = 89)	Female (n = 159)
Mean age ± SD	53.6 ± 17.2	57.8 ± 17.9	51.2 ± 16.3
Mean SDS ^1^ score ± SD	38.3 ± 7.4	37.8 ± 7.4	38.6 ± 7.4
# individuals with SDS < 40	143	48	95
# individuals with SDS 40–47	85	37	48
# individuals with SDS 48–55	23	7	16
# individuals with SDS > 55	2	0	2

^1^ SDS: Self-rating Depression Scale. Note: Discrepancies between the number of individuals within specific SDS score bands and total numbers are due to missing SDS data from certain participants. N = 254 includes all individuals with complete SDS total score data, regardless of genotype (including AA).

**Table 2:** Smoking and drinking rates across sex and genotype subgroups.

Sex	Genotype	# Smokers ^1^	# Drinkers ^2^	Alcohol Level ^3^ (Mean ± SD)
Male	GG; ss	1/11	5/11	1.64 ± 0.92
Male	GA; ss	2/10	4/10	1.8 ± 1.14
Male	GG; sl	4/38	25/38	2.39 ± 1.31
Male	GA; sl	0/20	11/20	1.95 ± 1.15
Male	GG; ll	2/15	10/15	2.13 ± 1.13
Male	GA; ll	0/4	2/4	1.75 ± 0.96
Female	GG; ss	1/22	10/22	1.71 ± 0.85
Female	GA; ss	1/8	3/8	1.38 ± 0.52
Female	GG; sl	0/57	34/57	1.79 ± 0.8
Female	GA; sl	0/25	16/25	2.0 ± 1.0
Female	GG; ll	3/34	17/34	1.71 ± 0.87
Female	GA; ll	0/16	13/16	2.38 ± 1.02

^1^ ‘Smoker’ defined as an answer of ‘yes’ to the question “Do you smoke?”. ^2^ ‘Drinker’ defined as any answer > 0 in response to the question “On average how many standard alcoholic drinks do you have per week?”. ^3^ ‘Alcohol level’ defined from 1 to 5 in response to the question “On average how many standard alcoholic drinks do you have each week?” where 1 = 0, 2 = 1 to 5, 3 = 6 to 10, 4 = 11 to 15, and 5 = more than 15.

**Table 3:** BMI across sex and genotype subgroups.

Sex	Genotype	BMI (Mean ± SD) ^1^
Male	GG; ss	25.87 ± 2.89
Male	GA; ss	27.46 ± 3.68
Male	GG; sl	28.76 ± 7.06
Male	GA; sl	28.64 ± 5.84
Male	GG; ll	27.76 ± 4.28
Male	GA; ll	26.04 ± 5.01
Female	GG; ss	30.62 ± 10.47
Female	GA; ss	28.07 ± 6.66
Female	GG; sl	27.42 ± 7.06
Female	GA; sl	27.03 ± 5.35
Female	GG; ll	27.25 ± 6.42
Female	GA; ll	28.89 ± 6.38

^1^ BMI (body mass index) calculated as weight (kg)/height (m)^2^.

## Data Availability

All data supporting the findings are within this paper and Appendix A.

## Abbreviations

The following abbreviations are used in this manuscript:

5-HTTLPR	Serotonin-transporter-linked promoter region
A	Adenine
ANCOVA	Analysis of covariance
BDNF	Brain-derived neurotrophic factor
BMI	Body mass index
C	Cytosine
CDRISC	Connor–Davidson resilience scale
DNA	Deoxyribonucleic acid
DSM-5-TR	*Diagnostic and statistical manual of mental disorders, 5th edition, text revision*
ELISA	Enzyme-linked immunosorbent assay
G	Guanine
HPA	Hypothalamic-pituitary-adrenal
HWE	Hardy–Weinberg equilibrium
PCR	Polymerase chain reaction
SAS	Zung Self-rating Anxiety Scale
SDS	Zung Self-rating Depression Scale
T	Thymine
UV	Ultraviolet

## Appendix A

**Table A1:** Raw scores for all psychometric measures used in this study.

Sex	Genotype	# ^1^	SAS ^2^	Cortisol	SDS ^3^ (D ^4^)	SDS (S ^5^)	SDS (C ^6^)	SDS (A ^7^)	CDRISC ^8^
Male	GG; ss	11	35.0 ± 6.9	20.2 ± 9.2	1.8 ± 0.6	1.5 ± 0.5	2.4 ± 1.1	2.7 ± 0.6	61.1 ± 20.0
	GA; ss	10	30.0 ± 8.3	21.7 ± 3.0	1.7 ± 0.6	1.4 ± 0.5	2.7 ± 1.1	2.2 ± 0.7	76.1 ± 17.0
	AA; ss	0	no data	no data	no data	no data	no data	no data	no data
	GG; sl	38	31.9 ± 8.1	18.5 ± 9.1	1.7 ± 0.6	1.5 ± 0.5	2.4 ± 1.0	2.6 ± 0.6	73.1 ± 15.8
	GA; sl	20	31.7 ± 9.8	19.7 ± 9.2	1.5 ± 0.6	1.5 ± 0.4	2.1 ± 1.1	2.5 ± 0.5	78.3 ± 11.8
	AA; sl	2	28.5 ± 4.9	16.1 ± 0.9	1.4 ± 0.6	1.4 ± 0.1	3.3 ± 1.1	2.3 ± 0.7	86.5 ± 21.9
	GG; ll	15	32.1 ± 6.3	19.3 ± 7.9	1.9 ± 0.4	1.5 ± 0.4	2.2 ± 1.5	2.2 ± 0.5	69.4 ± 12.7
	GA; ll	4	38.8 ± 4.6	22.7 ± 5.4	1.9 ± 0.3	1.5 ± 0.3	1.8 ± 1.5	2.9 ± 0.6	68.0 ± 27.6
	AA; ll	1	31.0	28.3	1.7	1.3	4	2.3	53.0
Female	GG; ss	22	33.7 ± 8.4	17.0 ± 9.0	1.7 ± 0.6	1.6 ± 0.4	2.3 ± 1.2	2.4 ± 0.5	74.8 ± 14.7
	GA; ss	8	40.9 ± 8.7	18.4 ± 9.2	1.3 ± 0.3	1.8 ± 0.7	1.6 ± 1.1	2.6 ± 0.3	74.1 ± 17.0
	AA; ss	1	39.0	16.1	2.3	1.8	1	2.8	66.0
	GG; sl	57	35.4 ± 8.4	19.5 ± 8.7	1.6 ± 0.5	1.6 ± 0.4	2.1 ± 1.2	2.4 ± 0.6	74.6 ± 15.4
	GA; sl	25	33.0 ± 8.8	16.9 ± 9.5	1.7 ± 0.6	1.5 ± 0.6	2.1 ± 1.2	2.7 ± 0.5	73.4 ± 14.9
	AA; sl	6	43.8 ± 9.5	29.1 ± 4.2	2.2 ± 0.5	2.0 ± 0.2	2.0 ± 1.2	2.1 ± 0.7	61.0 ± 19.2
	GG; ll	34	36.9 ± 9.1	20.5 ± 7.6	1.7 ± 0.7	1.7 ± 0.6	2.5 ± 1.2	2.6 ± 0.7	69.6 ± 17.3
	GA; ll	16	32.5 ± 8.2	18.2 ± 8.2	1.5 ± 0.5	1.6 ± 0.4	2.7 ± 1.3	2.4 ± 0.6	69.9 ± 15.3
	AA; ll	1	50	24.6	2.2	2.2	1	2.5	49.0

^1^ #: number of participants; ^2^ SAS: Zung Self-rating Anxiety Scale; ^3^ SDS: Zung Self-rating Depression Scale; ^4^ D: Depressed mood subscale; ^5^ S: Somatic depression subscale; ^6^ C: Cognitive depression subscale; ^7^ A: Anhedonic depression subscale; ^8^ CDRISC: Connor–Davidson Resilience Scale. Note: raw data is displayed as mean ± SD except for cells where n = 1 for AA individuals, where the data for the individual is displayed instead. Please note that the displayed values for all AA individuals have been included here for the purpose of transparency despite limited statistical value. AA individuals were excluded from the main formal analysis of this study.

**Table A2:** Complete omnibus ANCOVA results.

SAS ^1^			
Term	F	*p*	η^2^p
Sex	3.081	0.0806	0.0137
BDNF genotype	0.825	0.3647	0.0037
5-HTTLPR genotype	0.246	0.7820	0.0022
Sex × BDNF genotype	0.183	0.6688	0.0008
Sex × 5-HTTLPR genotype	0.013	0.9872	0.0001
BDNF genotype × 5-HTTLPR genotype	0.330	0.7190	0.0030
Sex × BDNF genotype × 5-HTTLPR genotype	4.497	0.0122	0.0389
Age	13.049	0.0004	0.0555
SDS ^2^ (D ^3^)			
Term	F	*p*	η^2^p
Sex	3.301	0.0706	0.0143
BDNF genotype	1.835	0.1769	0.0080
5-HTTLPR genotype	0.115	0.8914	0.0010
Sex × BDNF genotype	0.001	0.9783	<0.0001
Sex × 5-HTTLPR genotype	1.365	0.2574	0.0119
BDNF genotype × 5-HTTLPR genotype	1.510	0.2230	0.0131
Sex × BDNF genotype × 5-HTTLPR genotype	1.048	0.3522	0.0092
Age	13.491	0.0003	0.0561
SDS (S ^4^)			
Term	F	*p*	η^2^p
Sex	1.708	0.1925	0.0075
BDNF genotype	0.015	0.9028	0.0001
5-HTTLPR genotype	0.248	0.7806	0.0022
Sex × BDNF genotype	0.053	0.8189	0.0002
Sex × 5-HTTLPR genotype	0.839	0.4335	0.0073
BDNF genotype × 5-HTTLPR genotype	0.368	0.6928	0.0032
Sex × BDNF genotype × 5-HTTLPR genotype	0.162	0.8501	0.0014
Age	21.583	<0.0001	0.0868
SDS (C ^5^)			
Term	F	*p*	η^2^p
Sex	0.007	0.9346	<0.0001
BDNF genotype	0.410	0.5225	0.0018
5-HTTLPR genotype	2.322	0.1004	0.0200
Sex × BDNF genotype	0.096	0.7570	0.0004
Sex × 5-HTTLPR genotype	1.655	0.1935	0.0144
BDNF genotype × 5-HTTLPR genotype	0.183	0.8332	0.0016
Sex × BDNF genotype × 5-HTTLPR genotype	1.190	0.3061	0.0104
Age	4.195	0.0417	0.0181
SDS (A ^6^)			
Term	F	*p*	η^2^p
Sex	0.382	0.5374	0.0017
BDNF genotype	0.689	0.4073	0.0030
5-HTTLPR genotype	0.461	0.6310	0.0041
Sex × BDNF genotype	2.037	0.1549	0.0089
Sex × 5-HTTLPR genotype	0.229	0.7952	0.0020
BDNF genotype × 5-HTTLPR genotype	0.443	0.6424	0.0039
Sex × BDNF genotype × 5-HTTLPR genotype	5.755	0.0036	0.0485
Age	11.488	0.0008	0.0484
CDRISC ^7^			
Term	F	*p*	η^2^p
Sex	0.960	0.3282	0.0042
BDNF genotype	1.095	0.2964	0.0048
5-HTTLPR genotype	1.654	0.1937	0.0144
Sex × BDNF genotype	1.338	0.2485	0.0059
Sex × 5-HTTLPR genotype	2.078	0.1275	0.0180
BDNF genotype × 5-HTTLPR genotype	0.852	0.4279	0.0075
Sex × BDNF genotype × 5-HTTLPR genotype	0.577	0.5623	0.0051
Age	13.257	0.0003	0.0522
Morning Cortisol			
Term	F	*p*	η^2^p
Sex	0.422	0.5166	0.0020
BDNF genotype	0.459	0.4989	0.0021
5-HTTLPR genotype	1.184	0.3082	0.0109
Sex × BDNF genotype	3.554	0.0607	0.0163
Sex × 5-HTTLPR genotype	0.453	0.6366	0.0042
BDNF genotype × 5-HTTLPR genotype	0.638	0.5292	0.0059
Sex × BDNF genotype × 5-HTTLPR genotype	0.144	0.8659	0.0013
Age	0.001	0.9781	<0.0001
Body Mass Index			
Term	F	*p*	η^2^p
Sex	0.020	0.8872	0.0001
BDNF genotype	0.003	0.9567	<0.0001
5-HTTLPR genotype	0.284	0.7529	0.0029
Sex × BDNF genotype	0.009	0.9560	<0.0001
Sex × 5-HTTLPR genotype	1.794	0.1691	0.0182
BDNF genotype × 5-HTTLPR genotype	0.142	0.8677	0.0015
Sex × BDNF genotype × 5-HTTLPR genotype	0.477	0.6211	0.0049
Age	1.209	0.2729	0.0062

^1^ SAS: Zung Self-rating Anxiety Scale; ^2^ SDS: Zung Self-rating Depression Scale; ^3^ D: Depressed mood subscale; ^4^ S: Somatic depression subscale; ^5^ C: Cognitive depression subscale; ^6^ A: Anhedonic depression subscale; ^7^ CDRISC: Connor–Davidson Resilience Scale.

**Table A3:** FDR-adjusted *p*-values for all stratified tests.

Global			
Sex × BDNF test (global)			
Assessment stratum	5-HTTLPR genotype stratum	Nominal *p*-value	FDR-corrected *p*-value
SAS ^1^	ss	0.0289	0.3001
SAS	sl	0.4410	0.6789
SAS	ll	0.0612	0.3001
SDS ^2^ (D ^3^)	ss	0.1720	0.5709
SDS (D)	sl	0.3767	0.6789
SDS (D)	ll	0.7756	0.8461
SDS (S ^4^)	ss	0.6563	0.7876
SDS (S)	sl	0.6079	0.7679
SDS (S)	ll	0.9493	0.9493
SDS (C ^5^)	ss	0.2509	0.6690
SDS (C)	sl	0.5478	0.7304
SDS (C)	ll	0.4270	0.6789
SDS (A ^6^)	ss	0.0415	0.3001
SDS (A)	sl	0.0625	0.3001
SDS (A)	ll	0.0485	0.3001
CDRISC ^7^	ss	0.3464	0.6789
CDRISC	sl	0.3172	0.6789
CDRISC	ll	0.7246	0.8282
Morning Cortisol	ss	0.4809	0.6789
Morning Cortisol	sl	0.1291	0.5166
Morning Cortisol	ll	0.1903	0.5709
Body Mass Index	ss	0.4688	0.6789
Body Mass Index	sl	0.9374	0.9493
Body Mass Index	ll	0.4582	0.6789
Within-Stratum			
Sex × BDNF test (ss stratum)			
Assessment stratum	5-HTTLPR genotype stratum	Nominal *p*-value	FDR-corrected *p*-value
SAS	ss	0.0289	0.1658
SDS (D)	ss	0.1720	0.4586
SDS (S)	ss	0.6563	0.6563
SDS (C)	ss	0.2509	0.5018
SDS (A)	ss	0.0415	0.1658
CDRISC	ss	0.3464	0.5496
Morning Cortisol	ss	0.4809	0.5496
Body Mass Index	ss	0.4688	0.5496
Sex × BDNF test (sl stratum)			
Assessment stratum	5-HTTLPR genotype stratum	Nominal *p*-value	FDR-corrected *p*-value
SAS	sl	0.4410	0.6948
SDS (D)	sl	0.3767	0.6948
SDS (S)	sl	0.6079	0.6948
SDS (C)	sl	0.5478	0.6948
SDS (A)	sl	0.0625	0.5002
CDRISC	sl	0.3172	0.6948
Morning Cortisol	sl	0.1291	0.5166
Body Mass Index	sl	0.9374	0.9374
Sex × BDNF test (ll stratum)			
Assessment stratum	5-HTTLPR genotype stratum	Nominal *p*-value	FDR-corrected *p*-value
SAS	ll	0.0612	0.2450
SDS (D0	ll	0.7756	0.8864
SDS (S)	ll	0.9493	0.9493
SDS (C)	ll	0.4270	0.7332
SDS (A)	ll	0.0485	0.2450
CDRISC	ll	0.4270	0.8864
Morning Cortisol	ll	0.1903	0.5075
Body Mass Index	ll	0.4582	0.7332

^1^ SAS: Zung Self-rating Anxiety Scale; ^2^ SDS: Zung Self-rating Depression Scale; ^3^ D: Depressed mood subscale; ^4^ S: Somatic depression subscale; ^5^ C: Cognitive depression subscale; ^6^ A: Anhedonic depression subscale; ^7^ CDRISC: Connor–Davidson Resilience Scale.
